# Analysis of Factors Affecting the Astigmatic Correction Outcomes of Keratorefractive Lenticule Extraction Surgery

**DOI:** 10.3390/jcm14144850

**Published:** 2025-07-08

**Authors:** Jiping Xu, Manli Liu, Quan Liu

**Affiliations:** State Key Laboratory of Ophthalmology, Zhongshan Ophthalmic Center, Sun Yat-sen University, Guangdong Provincial Key Laboratory of Ophthalmology and Visual Science, Guangdong Provincial Clinical Research Center for Ocular Diseases, Guangzhou 510060, China; xujiping@gzzoc.com (J.X.); liumanli@gzzoc.com (M.L.)

**Keywords:** keratorefractive lenticule extraction, astigmatic correction, sphere-to-cylinder ratio, preoperative cylinder

## Abstract

**Background:** Keratorefractive Lenticule Extraction (KLEx) is an emerging flap-free surgical technique for the correction of myopic astigmatism. However, postoperative astigmatic accuracy remains variable. This study aimed to identify clinical and surgical factors that influence the outcomes of astigmatic correction following KLEx. **Methods:** A total of 98 patients with myopic astigmatism underwent KLEx. Manifest refraction was evaluated at three months postoperatively. Astigmatic outcomes were assessed using Alpins vector analysis. Multivariate linear and logistic regression models were used to determine associations between preoperative and intraoperative variables—such as age, eye laterality, tear film quality, sphere-to-cylinder ratio, and preoperative cylinder—and astigmatic correction parameters, including residual cylinder, correction index, magnitude of error, and angle of error. **Results:** Older age was associated with larger residual cylinder and angle of error. Eye laterality and tear film quality significantly influenced correction accuracy. A higher sphere-to-cylinder ratio and preoperative cylinder were also predictive of astigmatic correction performance. **Conclusions:** The accuracy of astigmatic correction in KLEx is significantly influenced by multiple clinical and surgical factors. Awareness of these predictors may guide surgical planning and improve refractive outcomes.

## 1. Introduction

Keratorefractive Lenticule Extraction (KLEx) is a flap-free and minimally invasive lamellar corneal refractive surgery technique assisted with a femtosecond laser [1]. KLEx has exhibited favorable efficacy, safety, predictability, and stability in the long term or short term [1,2]. Compared to laser in situ keratomileusis (LASIK), KLEx improves corneal biomechanical stability, leads to fewer dry eye symptoms, reduces postoperative higher-order aberrations, and minimizes inflammation after surgery [3]. However, regarding astigmatism correction, a greater general tendency toward under-correction has been reported with attempted KLEx treatments [4,5,6,7]. Corneal astigmatism can lead to low contrast sensitivity and poor visual function [8]. Visual quality after refractive surgery is the main concern for the patient and the surgeon. Residual astigmatism is one of the major factors affecting patients’ satisfaction with visual quality after KLEx, and this factor affects patients’ decisions to undergo another enhancement surgery [9,10,11]. Therefore, factors that may improve the accuracy of KLEx astigmatism correction need to be confirmed by further studies.

The VisuMax femtosecond laser system (Carl Zeiss Meditec, Jena, Germany) cannot automatically correct the eye cyclotorsion when the patients lie flat. This was believed to be one of the main factors contributing to unsatisfactory astigmatic correction with KLEx [12,13]. However, our previous study indicated that cyclotorsion during KLEx surgery did not affect astigmatic correction or postoperative visual quality [14]. In addition, cyclotorsion is a controllable factor that can be compensated manually by preoperative corneal marking and intraoperative rotation of the cone [12,14,15,16]. It was also thought that imprecise optical zone centration during KLEx may affect the outcome of astigmatic correction [17,18]. However, in a KLEx prospective study, no significant association was found between decentration and residual astigmatism among high and low astigmatism groups [19]. Furthermore, decentration can be controlled using the subjective patient fixation method or the triple marking centration method [20].

In addition to the two widely studied factors mentioned above, several other factors were reported to affect the accuracy of astigmatic correction with KLEx. A literature review has summarized various factors that may affect the precision of astigmatism correction, including the magnitude of preoperative astigmatism, angle Kappa, anterior corneal curvature, ocular residual astigmatism, opening incision position, with-the-rule or against-the-rule preoperative astigmatism, the method and the completeness of lenticule extraction, and the learning curve of the operator [18]. However, many important preoperative features that affect the outcome of astigmatism correction after KLEx have not been identified and reported. For example, none of the previous studies have investigated the effects of preoperative SE, axial deviation between K1 and cylinder, and central corneal thickness or optical diameter on astigmatic correction outcomes with KLEx. In the present study, we systematically analyzed the potential factors that may affect the astigmatic correction outcomes after KLEx.

## 2. Patients and Methods

### 2.1. Patients

**Figure 1:**
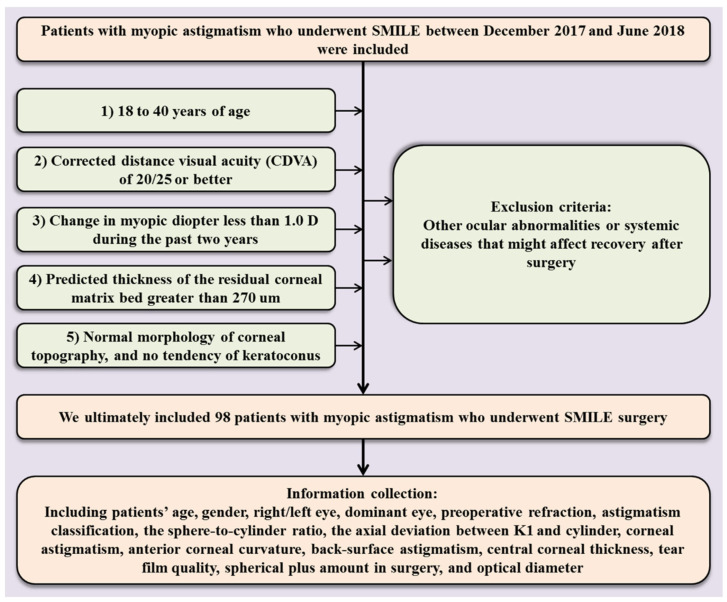
Sample Screening and Clinical Information Collection Process.

This prospective analytical study was approved by the Ethics Committee of Zhongshan Ophthalmic Center of Sun Yat-sen University and followed the tenets of the Declaration of Helsinki. Informed consent was signed by all patients before the KLEx surgery. We included 98 patients with myopic astigmatism who underwent KLEx surgery between December 2017 and June 2018. The inclusion criteria were as follows: (1) 18 to 40 years of age; (2) corrected distance visual acuity (CDVA) of 20/25 or better; (3) change in myopic diopter less than 1.0 D during the past two years; (4) predicted thickness of the residual corneal matrix bed greater than 270 um; and (5) normal morphology of corneal topography and no tendency of keratoconus. The exclusion criteria were as follows: other ocular abnormalities or systemic diseases that might affect recovery after surgery (Figure 1). All patients were of East Asian ethnicity (Han Chinese) and were recruited from Zhongshan Ophthalmic Center, a tertiary eye care center located in Southern China. As the prevalence and characteristics of myopic astigmatism may vary across populations, this demographic context should be considered when interpreting the results.

### 2.2. Surgical Methods

All surgeries were performed using the 500 kHz VisuMax femtosecond laser platform (Carl Zeiss Meditec, Jena, Germany) and were conducted by a single experienced surgeon (Q.L.). The surgical parameters used for all cases were as follows: cut energy of 110–120 nJ with spot distance of 4.5 µm, optical zone of 5.8 to 7.2 mm with a transition zone of 0.1 mm, cap thickness of 110 to 120 µm with diameter of 7.4 to 7.6 mm, incision width of 2 mm with position at 130° direction.

### 2.3. Data Collection

Manifest refractions were measured 3 months after surgery, and astigmatic outcomes were estimated using the vector analysis method of Alpins [21,22]. Manifest refractions with a vertex distance of 12 mm were converted from the spectacle plane to the corneal plane. We collected potential influence factors, including patients’ age, gender, right/left eye, dominant eye, preoperative refraction, and astigmatism classification, which includes the with-the-rule (WTR) astigmatism group, the against-the-rule (ATR) astigmatism group, and the oblique astigmatism group. Astigmatism was determined to be WTR astigmatism when the steepest meridian in the anterior cornea was within ±30 degrees of the vertical axis. Astigmatism where the steepest meridian was ± 30 degrees from the horizontal axis was considered ATR astigmatism. All other astigmatisms are considered oblique astigmatism, the sphere-to-cylinder ratio, the axial deviation between K1 and cylinder, corneal astigmatism, anterior corneal curvature, back-surface astigmatism, central corneal thickness, tear film quality, spherical plus amount in surgery, and optical diameter. We assessed their effects on postoperative parameters. Among these factors, axial deviation between K1 and cylinder (Degree), corneal astigmatism (D), anterior corneal curvature, corneal back surface astigmatism, and central corneal thickness were obtained from the Pentacam (Oculus Optikgeräte GmbH, Wetzlar, Germany). The tear film quality was measured using the Optical Quality Analysis System (OQAS-II, Visiometrics, Barcelona, Spain), with a smaller value representing better tear film stability. To avoid the bias of correlation, we randomly selected one eye of each patient for analysis. Although myopic astigmatism duration could theoretically influence corneal adaptation and surgical response, it was not included as a variable in this study because the onset time was typically unknown. Most cases of regular astigmatism are congenital or develop early in life, and patients are usually unable to recall when it began. Moreover, clinical records rarely document the duration of astigmatism prior to refractive surgery. Therefore, this information could not be reliably obtained. Baseline demographic and ocular parameters were collected and summarized (Table 1).

### 2.4. Data Analysis

Statistical analyses were performed using SPSS version 20 (SPSS Inc., Chicago, IL, USA). A normality test and homogeneity test of variables were conducted before analysis. Non-normally distributed continuous variables were converted to classified variables or rank variables where necessary. Bivariate correlation analyses were conducted between the influencing factors and postoperative parameters. A Pearson correlation analysis was performed for normally distributed variables, and Spearman rank correlation analysis was conducted for classified variables or non-normally distributed variables. Chi-square test and ANOVA analysis were performed when necessary. Risk factor variables with *p* < 0.2 in univariate analyses or those with clinical interest were included in multivariate models. Stepwise regression with inclusion criteria *p* < 0.05 and exclusion criteria *p* > 0.10 was used to select the optimal variable combination for multivariate linear regression analysis. Multivariate logistic regressions were also conducted when the dependent variables were classification variables or rank variables. Gender was included as one of the covariates in both univariate and multivariate analyses to evaluate its potential influence on astigmatic correction outcomes. *p* values less than 0.05 were considered to be statistically significant. Odds ratios for independent influencing factors are presented with their 95% confidence interval (CI). Vectorial analysis was visualized using quad polar plots according to the method proposed by Randleman and Reinstein [23].

## 3. Results

### 3.1. Basic Data

**Figure 2:**
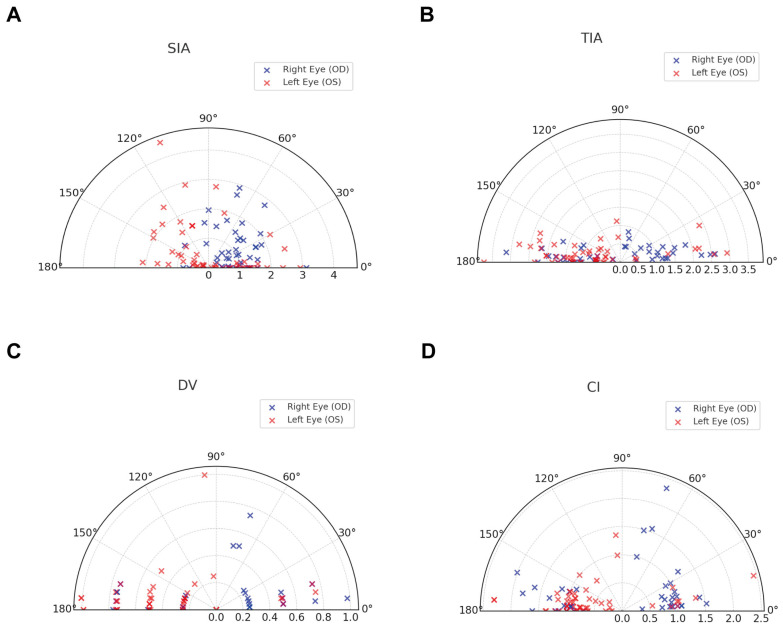
Quad polar plots displaying the vectorial parameters of astigmatic correction. Each dot represents an individual eye. Black arrows represent the mean vector of each parameter. (**A**) Surgically induced astigmatism (SIA) plotted using postoperative axis; (**B**) target-induced astigmatism (TIA), (**C**) difference vector (DV), and (**D**) correction index (CI) plotted using preoperative axis.

This study included 98 eyes of 98 patients with myopic astigmatism for analysis (65 females and 33 males; 44 right eyes and 54 left eyes; 63 dominant eyes and 35 non-dominant eyes). Their mean preoperative cylinder was −1.52 ± 0.85 D (range: −4.25 to −0.25 D) (with the rule: 85 eyes; against the rule: 6 eyes; oblique: 7 eyes). The mean preoperative spherical equivalent refraction was −5.88 ± 1.82 D (range: −11.00 to −2.00 D). Other characteristics of patients and surgical parameters are shown in Table 1. Vector parameters for residual cylinder and astigmatism correction after 3 months of KLEx are shown in Table 2. There were 75 eyes that had residual cylinder (with the rule: 35 eyes; against the rule: 15 eyes; oblique: 25 eyes), and 23 eyes had residual cylinder more than −0.50 D. To further visualize the axis alignment and directional distribution of the vectors, a quad polar plot was constructed following the methodology proposed by Randleman and Reinstein [23] The polar display illustrates the angular relationships between the principal vectors and highlights systematic rotational trends or misalignments that may not be evident from numerical data alone (Figure 2). Together, the table and the polar plot provide a complementary understanding of the astigmatic correction performance in our KLEx-treated population.

### 3.2. Bivariate Analyses

The results of the bivariate analyses for potential predictors and astigmatic correction outcomes are summarized in Table 3. The residual cylinder 3 months after KLEx and the difference vector were affected only by patients’ age. The magnitude of error and the correction index differed between the left eye and the right eye. The index of success was affected by several factors, including patients’ age, preoperative cylinder refraction, the sphere-to-cylinder ratio, corneal astigmatism, and back-surface astigmatism. The patients’ age, preoperative cylinder refraction, and the sphere-to-cylinder ratio were also associated with the absolute value of the angle of error. Table 4 shows that postoperative astigmatism classification 3 months after KLEx was not affected by preoperative astigmatism classification. The residual cylinder against the rule was associated with worse tear film quality before the surgery.

### 3.3. Multivariate Linear Regression

Statistically significant results in multivariate linear regressions are shown in Table 5. The Astigmatism Correction Index was associated with the right eye, higher sphere-to-cylinder ratio, and poorer tear film quality. The magnitude of error was associated with the left eye, better tear film quality, and less spherical plus amount (0.678 D per D; *p* < 0.05).

### 3.4. Multivariate Logistic Regression

Multivariate logistic regression analyses revealed that a larger preoperative cylinder was independently associated with a larger index of success (>0.27) (odds ratio = 2.803, 95% CI = 1.560−5.036) and larger |angle of error| (>4°) (odds ratio = 2.010, 95% CI = 1.142−3.540) (Table 6). Older age was independently associated with larger |angle of error| (>4°) (odds ratio = 1.154, 95% CI = 1.035−1.288). Right eyes and preoperative astigmatism with the rule were more likely to be associated with the angle of error < 0° (Table 7). Other preoperative and surgical characteristics, including gender, preoperative spherical equivalent refraction, axial deviation between K1 and cylinder, anterior corneal curvature, central corneal thickness, and optical diameter, were not associated with any of the astigmatic correction parameters.

## 4. Discussions

In our study, KLEx presented excellent astigmatic correction outcomes, with the average magnitude of error being 0 and the average correction index being 1.03. The vector analysis outcomes of astigmatic correction were similar to or even better than previous studies [4,17,24,25]. Moreover, Jun et al. reported improved astigmatic outcomes with vector planning in SMILE, indicating that incorporating vector-based nomograms could enhance correction accuracy in KLEx, particularly for eyes with high preoperative cylinders [26]. However, the average residual astigmatism was −0.38 D, which was greater than the average residual SE (−0.12 D) in the present study. This means that the accuracy of individual astigmatic correction with KLEx needs improvement. Based on bivariate correlation analysis, we found that patients’ age was associated with several vector parameters of astigmatism correction. Older age resulted in more residual cylinder, a larger difference vector, a larger absolute value of angle of error, and a larger index of success. In our multivariate logistic regression analysis, older age was more likely to be associated with |angle of error| > 4°. These results suggest that it is harder to achieve accurate astigmatic correction with KLEx among older patients, whereas previous studies found that age has no significant effects on the outcomes of astigmatic correction [27]. Our conflicting results may be explained by age-related effects on uneven corneal wound healing [28,29] or corneal epithelial remodeling, which affected the postoperative refractive status. Alternatively, older age may be associated with unstable tear film, thereby affecting the precision of optometry before astigmatic correction [30]. Although the gender distribution in our sample was not balanced, statistical analyses showed that gender had no significant effect on residual cylinder, correction index, or angle of error. Therefore, the observed gender imbalance is unlikely to have confounded the results.

In the present study, left eyes were more likely to be astigmatic under-corrected than right eyes based on their association with the magnitude of the error and the correction index. Based on the logistic regression of angle of error, the cylinder axis of left eyes was more likely to contra-rotate after surgery than right eyes. Consistent with our study, previous studies also found that vector analysis results of astigmatic correction with KLEx differed in the left and right eyes. Ivarsen et al. [27] found significant counterclockwise torsion of the astigmatic correction axis in the left eyes but reported no torsion in the right eyes. Yildiz et al. [31] revealed that left eyes had preferable astigmatic correction outcomes compared to those achieved in the right eyes. In their study, the correction index and the index of success were significantly better in the left eyes compared to the right eyes. Although our study found that the left and the right eyes were differently associated with vector parameters, no significant differences were found between the left eyes and the right eyes. In light of these observed interocular differences, we further explored the astigmatic vector characteristics using quad polar plots (Figure 2). The plots revealed a greater angular dispersion of SIA and DV in left eyes compared to right eyes, suggesting a tendency for counterclockwise axis deviation, which may relate to eye dominance or head positioning during surgery. These visual trends complement our quantitative findings and are consistent with prior reports using the Alpins method.

According to our results, the smaller the preoperative cylinder, the larger the |angle of error| and the index of success, which were preferable to be 0. Consistent with our results, previous studies revealed that preoperative cylinder affects astigmatic correction with KLEx. We found that there were 47% of eyes with low preoperative cylinder (−0.75 D to −1.00 D) having an absolute value of angle of error of more than 15°. Possibly, the axial accuracy of optometry was lower in low astigmatism than in high astigmatism cases [5]. We also found that low preoperative astigmatism (s −0.5 D) was associated with astigmatic overcorrection after KLEx, while moderate-to-high astigmatism was more likely to result in under-correction [4]. In addition, higher preoperative astigmatism resulted in greater degrees of under-correction [4,5,17,24]. However, our results did not show any correlation between the preoperative cylinder and the magnitude of error or the correction index.

The axis of preoperative astigmatism in the with-the-rule cases was more likely to rotate clockwise after KLEx than preoperative astigmatism in the against-the-rule cases. Previous studies also found that the axis classification of preoperative astigmatism can affect astigmatic correction with KLEx. Ivarsen et al. [27] revealed that less under-correction of 0.35 D was induced in preoperative astigmatism against-the-rule cases than in astigmatism with-the-rule cases. Perez-Izquierdo et al. [32] found that when preoperative astigmatism was 1.50 D or greater, more under-correction occurred in the eyes of preoperative astigmatism with-the-rule cases than in the other two groups. In our additional group analysis, we found that preoperative astigmatism in against-the-rule cases was more likely to be over-corrected than in the other types (correction index in preoperative astigmatism with the rule, oblique, and against the rule was 1.01, 0.84, and 1.46, respectively, *p* = 0.016). It has been suggested that a small amount of residual astigmatism with-the-rule is beneficial [33]. Thus, astigmatism may be better to be over-corrected than under-corrected for eyes with preoperative astigmatism against-the-rule, and the nomogram of KLEx does not need any special adjustments for these cases.

Based on multivariate linear regression analyses, the correction index was positively associated with the ratio of sphere to cylinder rather than the preoperative cylinder itself. The higher the ratio, the larger the |angle of error| and the index of success, which were preferable to be 0. This means that the astigmatic correction of eyes with a high sphere-to-cylinder ratio (which means less proportion of cylinder in SE) is more likely to be overcorrected and have axial deviation. However, other studies did not report the association between astigmatic correction and the sphere-to-cylinder ratio. Therefore, it was hypothesized that previous results might have been influenced by the proportion of cylinder rather than the magnitude of the cylinder itself [4,5,17,24]. Therefore, in addition to the magnitude of the cylinder, the proportion of the cylinder should be considered when adjusting the KLEx nomogram.

Using bivariate correlation analyses, we found that higher corneal astigmatism and back-surface astigmatism were associated with a smaller index of success after KLEx. However, multivariate analysis revealed that corneal astigmatism and back-surface astigmatism were not significantly associated with the astigmatic outcomes. Previous studies also found that back-surface astigmatism did not affect astigmatic correction [27]. Herein, both anterior corneal astigmatism and back-surface astigmatism were highly correlated and included in subjective cylindrical refraction, which was the target treatment. Based on multivariate linear regression analyses for the correction index and the magnitude of error, a worse tear film quality was correlated with astigmatic overcorrection. This may explain why poor tear film quality was associated with significantly more residual astigmatism in against-the-rule cases. It was speculated that a poor quality tear film affects the stability of visual function and the accuracy of pre-operative subjective refraction, leading to overmeasurement of the target astigmatism [30]. Alternatively, the poor quality of tear film may affect tissue healing after KLEx, consequently affecting the predictability of astigmatic correction [34].

The spherical plus amount in KLEx surgery was found to determine the factors affecting the magnitude of error. Larger amounts of spherical plus may lead to the overcorrection of astigmatism, but this was not mentioned in previous studies. In addition, this study found that gender, preoperative spherical equivalent refraction, axial deviation between K1 and cylinder, anterior corneal curvature, central corneal thickness, and optical diameter did not affect the astigmatic correction with KLEx. Consistently, previous studies found that gender had no significant effect on the vector analysis outcomes [27]. In contrast, previous studies revealed that steeper corneal curvature was associated with SE under-correction after KLEx [35].

Higher ORA is associated with poorer vectorial and visual outcomes [36]. In our study, the average postoperative residual astigmatism was −0.38 D, and some patients may have elevated ORA, which may contribute to the observed variability in astigmatic correction accuracy. Although ORA (K_TR = K_R − K_T) did not show a statistically significant correlation in our analysis, its potential prognostic value is evident from the axis deviation (9.07 ± 12.25°), which is consistent with previous findings [36]. This consistency indicates ORA may still play a role in optimizing nomograms, and its lack of significance may reflect study limitations rather than a lack of clinical relevance. Future studies should explore with a larger dataset and refined methodology to better elucidate ORA’s potential prognostic value.

Many authors have recommended customizing the astigmatic correction nomogram of KLEx based on their results [5,27,32,37]. Our systematic multivariable analysis indicated that patients’ age, left or right eye, preoperative cylinder, classification of preoperative astigmatism, the sphere-to-cylinder ratio, and tear film quality should be considered when planning for astigmatic correction with KLEx surgery. However, based on the total sample, we still believe that personalized adjustments are needed rather than unified corrections. The astigmatic outcomes were simultaneously affected by various factors rather than a single factor. Therefore, these adjustments should not be solely determined based on the findings of individual research centers, but should be based on the existence of many non-uniform factors, such as optometry habits, surgical experience, and different surgical machines. Finally, artificial intelligence based on big data is expected to more efficiently optimize astigmatic correction nomogram for KLEx surgery.

## 5. Limitation

One of the limitations of this study was the small sample size. Particularly, there were few eyes with “against-the-rule” cases and oblique astigmatism cases. The recognition of some other influencing factors might have been limited by the small sample size. Furthermore, the unbalanced sample of preoperative astigmatism types might have limited their effect on outcomes. The relatively short follow-up time may be another limitation of our study. However, previous studies have shown no significant differences in cylindrical refraction between follow-up times, even 1 year after KLEx [4]. Furthermore, the physiological reasons behind these correlations deserve further studies. Additionally, all patients in this study were of East Asian descent (Han Chinese). As ethnic differences may influence the prevalence and characteristics of myopic astigmatism as well as the response to refractive surgery, the generalizability of our findings to other populations may be limited. Further studies involving more diverse ethnic cohorts are needed to broaden the applicability of these results.

## 6. Conclusions

The astigmatic outcomes of KLEx were simultaneously affected by various preoperative and surgical factors, including patients’ age, left or right eye side, preoperative cylinder, classification of preoperative astigmatism, the sphere-to-cylinder ratio, tear film quality, and spherical plus amount in surgery. Taking these factors into account may help surgeons optimize the refractive nomogram and improve the precision of astigmatic correction after KLEx.

## Figures and Tables

**Table 1:** Demographic and Preoperative Characteristics of the Study Subjects.

Variable	Mean ± SD/N(%)	Range
Female	65 (66.3%)	
Male	33 (33.7%)	
Left Eye	50 (51.0%)	
Right Eye	48 (49.0%)	
Age (years)	26.11 ± 4.44	19–39
Cylinder (D)	−1.52 ± 0.85	−4.25~−0.25
Spherical equivalent (D)	−5.88 ± 1.82	−11.00~−2.00
Sphere-to-cylinder ratio	4.77 ± 3.66	0.30~24.00
Axis deviation between K1 and cylinder (°)	9.07 ± 12.25	0.20~65.40
Corneal astigmatism (D)	−1.83 ± 0.72	−3.70~−0.40
Keratometry (D)	43.54 ± 1.22	39.70~46.90
Posterior corneal astigmatism (D)	0.46 ± 0.14	0.00~0.80
Central corneal thickness (µm)	544.43 ± 25.34	494.00~604.00
Tear film quality	0.75 ± 0.73	0.05~4.04
Spherical correction amount (D)	−0.51 ± 0.10	−0.75~−0.25
Optical zone diameter (mm)	6.63 ± 0.30	6.00~7.30

D = diopters.

**Table 2:** Residual cylinder and vector analysis of astigmatic correction at 3 months after KLEx.

Parameters	Mean ± SD	Range
Post cylinder (D)	−0.38 ± 0.30	−1.00 to 0.00
Post SE (D)	−0.12 ± 0.37	−1.00 to 0.88
Difference vector (D)	0.39 ± 0.30	0.00 to 1.01
Magnitude of error (D)	0.00 ± 0.34	−0.86 to 0.74
Angle of error (degrees)	−3.13 ± 13.39	−68.00 to 51.43
|Angle of error| (degrees)	7.84 ± 11.30	0.00 to 68.00
Correction index	1.03 ± 0.41	0.19 to 2.44
Index of success	0.41 ± 0.43	0.00 to 1.76

D = diopters.

**Table 3:** Bivariate correlation coefficients for potential predictors and astigmatic correction outcomes at 3 months after KLEx ^a^.

Predictors	Post Cylinder	Post SE	Post Astigmatism Classification	Difference Vector	Magnitude of Error	Angle of Error	|Angle of Error|	Correction Index	Index of Success
Age	−0.205 *	0.034	−0.158	0.206 *	−0.048	−0.024	0.303 **	0.073	0.250 *
Gender	0.090	−0.074	0.021	−0.069	−0.007	0.017	−0.040	−0.030	−0.018
Eye (right/left)	−0.041	0.082	−0.012	0.066	0.222 *	0.146	−0.040	−0.258 *	0.037
Eye (dominant/non-dominant)	0.019	−0.152	−0.074	−0.065	0.049	−0.107	−0.058	−0.068	0.003
Pre Cylinder	0.075	−0.083	−0.054	−0.087	−0.046	−0.002	0.271 **	−0.016	0.403 **
Pre SE	0.099	−0.049	−0.020	−0.085	0.059	0.107	−0.025	−0.062	−0.003
The ratio of sphere to cylinder	0.006	−0.045	−0.057	−0.042	−0.061	−0.056	0.333 **	0.003	0.404 **
Axial deviation between K1 and cylinder	0.148	−0.016	0.000	−0.125	−0.046	0.103	0.003	0.045	0.093
Corneal astigmatism	0.177	0.029	−0.030	−0.134	−0.100	−0.033	0.146	0.048	0.283 **
Anterior corneal curvature	−0.004	−0.146	−0.150	−0.051	0.073	0.132	0.034	−0.063	0.013
Back-surface astigmatism	−0.011	−0.125	0.039	−0.047	0.070	0.101	−0.150	−0.023	−0.322 **
Central corneal thickness	−0.033	−0.150	0.013	0.046	0.036	0.075	0.066	−0.002	0.052
Tear film quality	−0.123	0.068	0.067	0.114	−0.193	−0.065	0.002	0.167	0.043
Spherical plus amount in surgery	0.022	0.020	−0.181	0.015	0.180	0.169	−0.061	−0.161	−0.029
Optical diameter	0.074	0.047	−0.036	−0.089	0.014	0.075	−0.081	−0.037	−0.027

^a^ Pearson for normal distribution continuous variables; Spearman for non-normal distribution or classified variables; * *p* < 0.05; ** *p* < 0.01.

**Table 4:** Preoperative characteristics associated with post astigmatism classification at 3 months after KLEx.

Preoperative Characteristics	With the Rule (Post) (n = 35)	Against the Rule (Post) (n = 15)	Oblique (Post) (n = 25)	No Residual Cylinder (n = 23)	*p* Value
With the rule (n = 85)	31	13	22	19	
Against the rule (n = 6)	2	0	2	2	
Oblique (n = 7)	2	2	1	2	0.977 ^a^
Tear film quality (Mean ± SD)	0.58 ± 0.45	1.27 ± 0.67	0.69 ± 0.69	0.79 ± 0.92	0.041 ^b^

^a^ Chi-square test; ^b^ ANOVA analysis of variance.

**Table 5:** Multiple linear regression analyses for the correction index and the magnitude of error at 3 months after KLEx (meaningful coefficient value) ^a^.

	Correction Index	Magnitude of Error (D)
Eye (right/left)	−0.221 **	0.133 *
The ratio of sphere to cylinder	0.024 **	
Tear film quality	0.208 **	−0.167 **
Spherical plus amount in surgery (D)		0.678 *

^a^ Values are presented as β value; D = diopters; * *p* < 0.05; ** *p* < 0.01.

**Table 6:** Multiple logistic regression model of preoperative characteristics associated with the index of success and |angle of error| at 3 months after KLEx (meaningful coefficient value).

Characteristics	Odds Ratio	95% CI
Characteristics associated with index of success > 0.27		
Pre Cylinder	2.803 **	1.560~5.036
Characteristics associated with |angle of error| > 4°		
Age	1.154 *	1.035~1.288
Pre Cylinder	2.010 *	1.142~3.540

* *p* < 0.05; ** *p* < 0.01; CI = Confidence Interval.

**Table 7:** Multiple logistic regression model of preoperative characteristics associated with angle of error < 0 at 3 months after KLEx (meaningful coefficient value).

Characteristics	Odds Ratio	95% CI
Different eye side		
Right eye	1.00	
Left eye	0.334 *	0.138~0.810
Preoperative astigmatism classification		
With the rule	1.00	
Against the rule	0.056 *	0.005~0.685
Oblique	0.123	0.010~1.443

* *p* < 0.05; CI = Confidence Interval.

## Data Availability

All data generated or analyzed during this study are included in this published article.
